# Radon exposure delays the development of skin lesions in a mouse model of psoriasis

**DOI:** 10.3389/fimmu.2025.1638483

**Published:** 2025-11-18

**Authors:** Sylvie Lerchl, Julia Wiedemann, Andreas Maier, Franziska Papenfuß, Kristian Unger, Anna Brand, Björn E. Clausen, Claudia Fournier

**Affiliations:** 1 Biophysics Department, GSI Helmholtzzentrum für Schwerionenforschung GmbH, Darmstadt, Germany; 2 Department of Radiation Oncology, University Hospital, Ludwig-Maximilians-University (LMU) Munich, Munich, Germany; 3 Research Unit Translational Metabolic Oncology, Institute for Diabetes and Cancer, Helmholtz Zentrum München Deutsches Forschungszentrum für Gesundheit und Umwelt (GmbH), Munich, Germany; 4 Bavarian Cancer Research Center (BZKF), Munich, Germany; 5 Institute for Molecular Medicine, University Medical Center of the Johannes Gutenberg-University Mainz, Mainz, Germany; 6 Research Center for Immunotherapy (FZI), Paul Klein Center for Immune Intervention, University Medical Center of the Johannes Gutenberg-University Mainz, Mainz, Germany

**Keywords:** radon treatment, psoriasis, CD11c-IL-17A^ind/ind^ psoriasis mouse model, alpha-particle irradiation, immune modulation

## Abstract

**Introduction:**

The chronic inflammatory skin disease psoriasis vulgaris is characterized by itchy plaques, often accompanied by life-threatening comorbidities severely impairing the quality of life and cause high socioeconomic costs. Despite the known cancer risk, radon inhalation is used as a treatment for various chronic inflammatory diseases, including psoriasis. Knowledge about the underlying mechanism is scarce, largely due to the lack of suitable mouse models.

**Methods:**

Here, we used transgenic mice that spontaneously develop chronic psoriatic skin lesions inflicted by constitutive low-level IL-17A-production by CD11c^+^ cells (CD11c-IL-17A^ind/ind^ mice). Mice underwent single or multiple radon exposures under therapy relevant conditions and observed until plaque formation or predefined time points. Blood and tissue were collected for immunohistological analysis and immune phenotyping. Comprehensive transcriptome profiling of non-lesional skin was performed 3 days and 2 weeks after multiple radon exposures.

**Results and discussion:**

Following multiple radon exposures, plaque formation was significantly delayed in CD11c-IL-17A^ind/ind^ mice, although IL-17A concentrations were not reduced. Cellular and molecular analyses indicated transient immunosuppressive effects after radon exposure and, on the cellular level, pDCs were significantly reduced in lymph nodes. Comprehensive transcriptome profiling of non-lesional skin showed a different gene expression profile after radon exposure. Notably, at an early time point after exposure, the *Tbx21* gene, associated with psoriasis initiation, and at a late time point Tgfbr1were significantly downregulated, Furthermore, genes related to the suppression of inflammation and immune activation (Ccr6), for example Gata3 and others were upregulated. This points to immune modulation after radon exposure. In line with this, pathway enrichment analysis revealed immunosuppressive effects related to T cell regulation, similar to UV radiation-induced response.

**Conclusion:**

This study provides the first evidence of the efficacy of radon treatment, including the underlying mechanisms in a preclinical mouse model.

## 1 Introduction

Radon is a naturally occurring, radioactive noble gas that is released from rocks and soil and exerts its biological effectiveness mainly via the emission of alpha-particles (1–3). Despite the known cancer risk associated with any exposure to radon (4–6), it is used to treat chronic inflammatory disorders such as the skin disease psoriasis (3) and in spa therapy via radon-containing water or the inhalation of radon-containing air in so-called radon galleries (mines) (7).

Radon gas enters the body through inhalation or skin contact and subsequent diffusion, after which it is absorbed by the blood and distributed to the organs (3, 8–10).

The autoimmune-mediated, chronic inflammatory skin disease psoriasis can occur in various clinical phenotypes. The most prevalent form and the one analyzed here is psoriasis vulgaris (plaque psoriasis), which manifests as well-demarcated erythema with adherent silvery scales (11, 12). According to current knowledge, the pathomechanism can be divided into an initiation phase and a chronic state (13). In the initiation phase, autoantigens like the antimicrobial peptide LL-37 (14) or the melanocyte-derived protein Adamtsl5 (15) activate different types of dendritic cells (DCs). Plasmacytoid DCs (pDCs) are effector cells that initiate the inflammatory cascade by releasing interferon (IFN)-alpha, thereby activating antigen-presenting and interleukin (IL)-12 or IL-23 producing classical DCs (cDCs). IL-23 and IL-12 subsequently polarize T helper (Th) type 17 (Th17) and type 1 (Th1) cells, respectively (11, 12, 16). Among the pro-inflammatory cytokines released by Th17 and Th1 cells, IL-17A is a key stimulator for keratinocytes to produce pro-inflammatory cytokines, chemokines, and antimicrobial peptides. These soluble factors cause the chronicity of psoriasis via a positive feedback loop that attracts additional immune cells such as DCs, T cells, and neutrophiles (11–13, 17, 18). Furthermore, hyperproliferation and altered maturation of keratinocytes lead to acanthosis and parakeratosis (12).

Systemic exposure to radon is currently used to treat patients with psoriasis (3). During treatment, patients are inhaling radon-containing air in radon galleries (generally former mines). An inhalation therapy in radon galleries typically consists of 10 1-h lasting sessions within a period of 2–3 weeks. In the thermal gallery of Bad Gastein in Austria, patients are exposed, e.g., to an average radon activity concentration of 45 kBq/m^3^ at 37–41.5 °C and 70%–99% relative humidity per session (7). However, there is only anecdotal evidence of rapid and long-lasting symptom relief. Investigations into the mechanism of action of radon therapy have been hampered by the lack of suitable *in vivo* models (3). In line with patient data and particularly the central role of IL-17A in the pathogenesis of psoriatic skin lesions, we have developed CD11c-IL-17A^ind/ind^ homozygous transgenic mice. This mouse model constitutively produces low concentrations of IL-17A by CD11c^+^ cells that drive the development of psoriatic skin lesions from 8 weeks of age with 100% penetrance, exhibiting crucial aspects of the human disease such as chronicity (19). Here, we aimed to use these CD11c-IL-17A^ind/ind^ transgenic mice to determine the mitigating effects of radon exposure (20) *in vivo* and to explore the underlying cellular and molecular mechanisms of disease suppression.

## 2 Methods

### 2.1 Mouse model

Homozygous CD11c-IL-17A^ind/ind^ mice were obtained from the University Medical Center of the Johannes Gutenberg-University Mainz (19). These mice are characterized by low but sustained production of the transgenic cytokine IL-17A in CD11c^+^ DCs. CD11c^+^ DCs account for an average of 2%–3% of leukocytes in all murine tissue types, which roughly corresponds to the number of IL-17A-producing leukocytes in human psoriatic plaques. The overexpression of IL-17A in CD11c-IL-17A^ind/ind^ mice leads to the development—starting in homozygous mice at an age of approximately 8 weeks—of gradually progressing plaque psoriasis, which is very similar to human psoriasis vulgaris.

The animal experiment was approved by the regional council (Regierungspräsidium Darmstadt, approval number: DA17/1000) and conducted in accordance with the guidelines of Federation of European Laboratory Animal Science Associations (FELASA). Mice were housed in groups using open cages placed in a ventilated scantainer (Scanbur) maintaining standard conditions [20–22 °C; 55% relative humidity (RH); 12-h light–dark cycle]. Animals had *ad libitum* access to food and water and were kept in an enriched environment.

### 2.2 Radon exposure

CD11c-IL-17A^ind/ind^ mice were exposed to radon in a chamber especially designed for animal and cell experiments. Using this chamber, radon concentration and temperature/relative humidity could be reproducibly adjusted at 22.7 ± 0.5 °C and 50%–70% RH (20). Mice [8.5 weeks old (±3 days)] were randomly distributed in groups to be exposed 1h either once to 539.0 ± 2.8 kBq/m³ (*n*=10; 6 females, 4 males) or on 10 consecutive workdays to 39.2 ± 2.0 kBq/m³ (*n*=22; 9 females, 13 males) radon activity concentration. Sham-exposed animals (single exposure: *n*=10; 7 females, 3 males; multiple exposures: *n*=21; 8 females, 13 males) underwent the same procedure under similar conditions except that the radon activity concentration was 0.12 ± 0.04 kBq/m³, which is the normal background concentration in buildings. At the time points of exposure, none of the animals already showed plaques (score 4, see section 2.3). A few animals showed a skin score of 1 or 2 (dry or scaly skin) before the start of radon treatment. These animals were equally distributed among the groups, resulting in a similar overall score at the start of radon exposure.

### 2.3 Scoring

Considering that commonly used scores like PASI (Psoriasis Area and Severity Index) were not adequate for this purpose, we established a modified severity score from 1 to 6 (see **Supplementary Table S1**; **Supplementary Figure S1**). Scoring was conducted three times per week. A score of 4 was counted as plaque. To be considered as “plaque induction”, the plaque had to be observed at least 2× per week and persist for at least 2 weeks.

### 2.4 Sacrificing and sample collection

Mice were euthanized when they reached a score higher than 5.5 or at defined time points after radon exposure, by cervical dislocation under deep isoflurane inhalation anesthesia (5% isoflurane) following the FELASA and AVMA guidelines for the euthanasia of animals. Skin tissue was fixed in 4% PFA solution for histological analysis or stored in RNAlater for RNA sequencing. Spleen and lymph nodes (LNs) were collected for immunophenotyping.

### 2.5 Cytokine measurements in serum

Blood was drawn from the tail vein one or two times before and every week during and after radon treatment period, and after sacrificing the mice. The blood was incubated in clotting activation tubes (Vacuette^®^ Blood Collection Tubes, Greiner Bio-One GmbH) for 30min and centrifuged (1,500 ×*g*, 10min, RT), and serum was separated and frozen. IL-17A and IL-19 concentrations in serum were measured via enzyme-linked immunosorbent assay (ELISA) according to the manufacturer’s instructions (Uncoated ELISA-Kits, Invitrogen AG).

### 2.6 Histology

Tissue samples were processed and analyzed microscopically as previously described (21) (see **Supplementary Materials**). Antibody staining was performed for Ki67 (monoclonal, SP6, abcam), FOXP3 (monoclonal, FJK 16s, eBioscience), RORγT (monoclonal, AFKJS-9, Invitrogen AG), or Ly6g (monoclonal, EPR22909-135, abcam).

### 2.7 Immunophenotyping of LN and spleen

For staining, 2 × 10^6^ cells per sample of spleen and LN were used. The applied standard procedure is described in the **Supplementary Materials**. Staining was performed with the following antibodies:

DC panel: CD11c (PerCP, N418) and MHCII (APC, M5/114.15.2) from BioLegend, B220 (Pacific Blue, RA3-6B2) and CD8a (APC-Cy7, 53-6.7) from BD Pharmingen, and CD11b (PE, M1/70) from eBiosciences; T-cell panel: CD3e (PE-Cy7, 145-2C11), CD4 (Pacific Blue, RM4-5), and CD25 (PE, 7D4) from BD Pharmingen and CCR6 (PerCP-Cy5.5, 29-2L17). Gating strategies are shown in **Supplementary Figure S3**.

### 2.8 Transcriptome profiling by RNA sequencing

Whole RNA sequencing of skin samples was performed by Microsynth Seqlab GmbH. Tissue samples were removed from RNAlater and placed in tubes with 750 µL of RLTplus (Qiagen) with 10 µL/mL β-mercaptoethanol (Thermo Fisher) and 2-mm steel beads, followed by processing on an FP24 at speed 6 for 1min. Lysate (200 µL) was used for RNA isolation with the Qiagen RNeasy plus kit. The RNA was quantified with the RiboGreen (Thermo Fisher) assay, and qualitative analysis was carried out on an Agilent FragmentAnalyzer.

Raw read counts were imported into R prior to differential gene expression analysis using the R package DEseq2 (22). Only genes that had a total sum count of 10 times the number of samples were kept. Genes were considered differentially expressed if the unadjusted *p*-value was smaller than 0.05.

Gene set enrichment analysis (GSEA) was performed as described in the **Supplementary Materials**.

### 2.9 Statistical analysis

Results were plotted and statistically analyzed with GraphPad Prism 9.3.1 software, R, or the online tool venny2.1. Independent Kaplan–Meier plots were compared via the Log-rank (Mantel–Cox) test. Independent samples/groups were compared using unpaired *t*-test when the Shapiro–Wilk test indicated normal distribution or the Mann–Whitney *U* test when the Shapiro–Wilk test showed no normal distribution.

## 3 Results

### 3.1 Delayed onset of psoriatic plaque formation in CD11c-IL-17A^ind/ind^ mice after radon exposure

**Figure 1:**
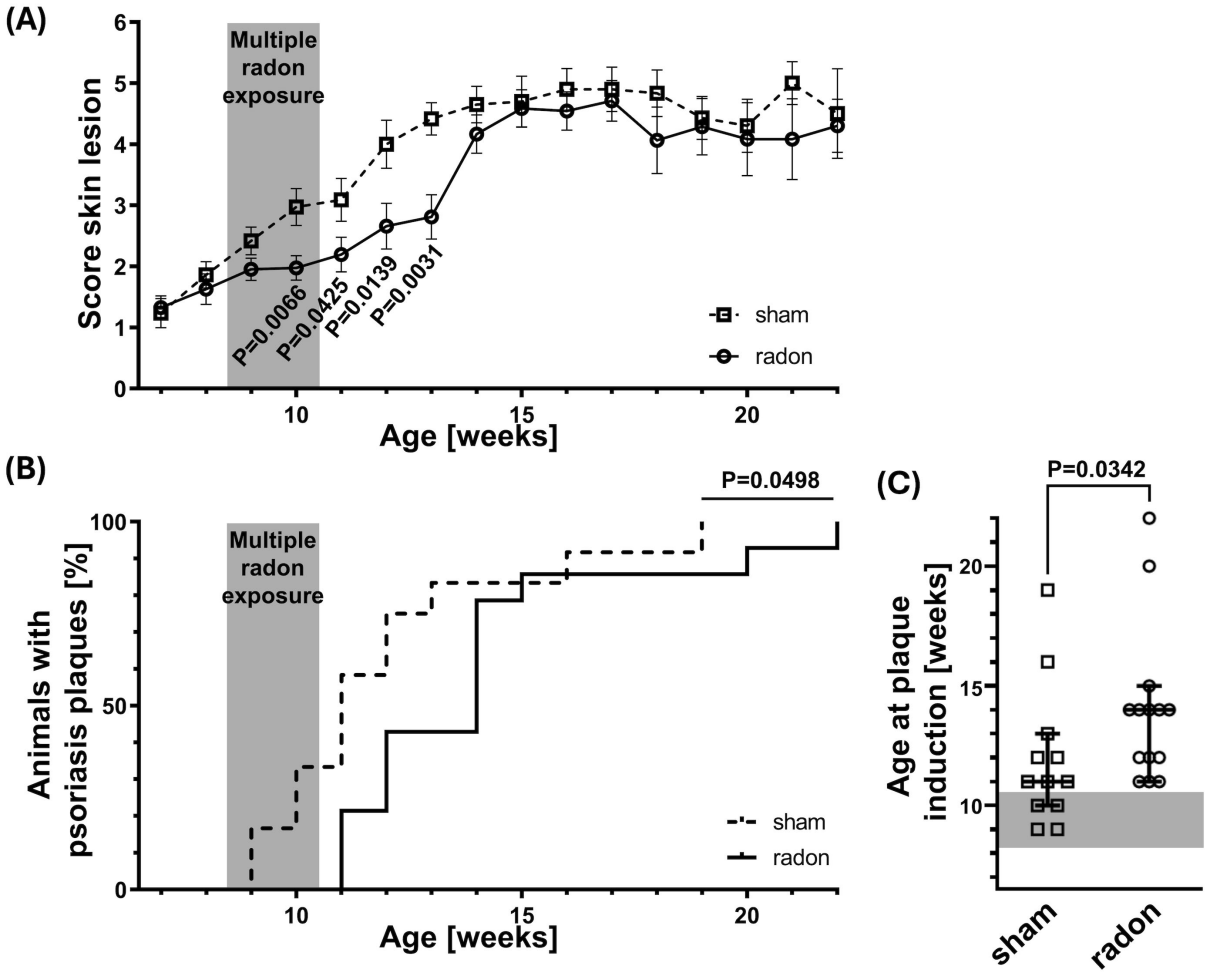
Multiple radon exposures delay the onset of plaque induction in CD11c-IL-17A^ind/ind^ mice. CD11c-IL-17A^ind/ind^ mice (8.5 weeks old ± 3 days) were exposed to a radon activity concentration of 39.2 ± 2.0 kBq/m³ or sham-treated in our radon chamber on 10 consecutive workdays (2 weeks, except weekends) for 1h each. The severity of skin reactions was examined three times per week, according to the score of 0–6, elucidated in **Supplementary Table S1**, reaching a score of 4 = time point of plaque induction. **(A)** Averaged score values of skin reactions over time for different ages in weeks; Mann–Whitney *U* test for each time point since the Shapiro–Wilk test showed no normal distribution for most datasets, *n* (sham) = 4–18, *n* (radon) = 5–20; mean values ± SEM. **(B)** Kaplan–Meier plot with percentage of animals showing characteristic psoriasis plaques (score ≥ 4) at different ages in weeks; Log-rank (Mantel–Cox) test, *n* (sham) = 12, *n* (radon) = 14. **(C)** Age in weeks at plaque induction (reaching score ≥ 4) for each mouse; Mann–Whitney *U* test since no normal distribution was indicated via the Shapiro–Wilk test, *n* (sham) = 12, *n* (radon) = 14; median with 95% confidence interval.

We previously reported that homozygous CD11c-IL-17A^ind/ind^ mice develop psoriatic skin lesions between the age of 8 and 18 weeks (19). To investigate if radon alleviates this phenotype, 8.5-week-old (±3 days) CD11c-IL-17A^ind/ind^ mice received total radon activity concentrations comparable to those in a radon gallery where patients are treated. Radon was administered either in a single exposure for 1h or—similar to patient treatment—in 10 consecutive 1-h exposures each over a period of 2 weeks by using a specially designed radon chamber (20). Before, during, and for several weeks after treatment, the severity of skin reactions in radon- and sham-exposed mice was scored (**Supplementary Table S1**). As depicted in **Figure 1A**, multiple radon exposures significantly reduced the mean severity score of skin reactions by 1–1.6 units as compared to sham-exposed control animals, starting from the second week of treatment and up to 3 weeks after the last session. In addition, the time point of full plaque induction (attaining a score of 4) was significantly delayed by 2–3 weeks (**Figure 1B**), postponing the median age of plaque development from 11 weeks in the sham-exposed group to 14 weeks in multiply radon-exposed mice (**Figure 1C**). Single radon exposures with a comparable total radon activity concentration showed a slightly delayed onset of plaque induction, which is not significant (**Supplementary Figure S2**). In conclusion, these data indicate that repeated exposures to radon result in a delayed development of psoriatic skin lesions in CD11c-IL-17A^ind/ind^ mice.

### 3.2 No systemic effects on serum concentrations of IL-17A and IL-19 in radon-treated CD11c-IL-17A^ind/ind^ mice

To check whether the significant improvement in skin reactions of CD11c-IL-17A^ind/ind^ mice was facilitated by a systemic change of key cytokines after multiple radon exposures, we measured the serum concentrations of two central cytokines of the pathogenic IL-23/IL-17 axis, namely, IL-17A and IL-19, at different time points (13, 23).

**Figure 2:**
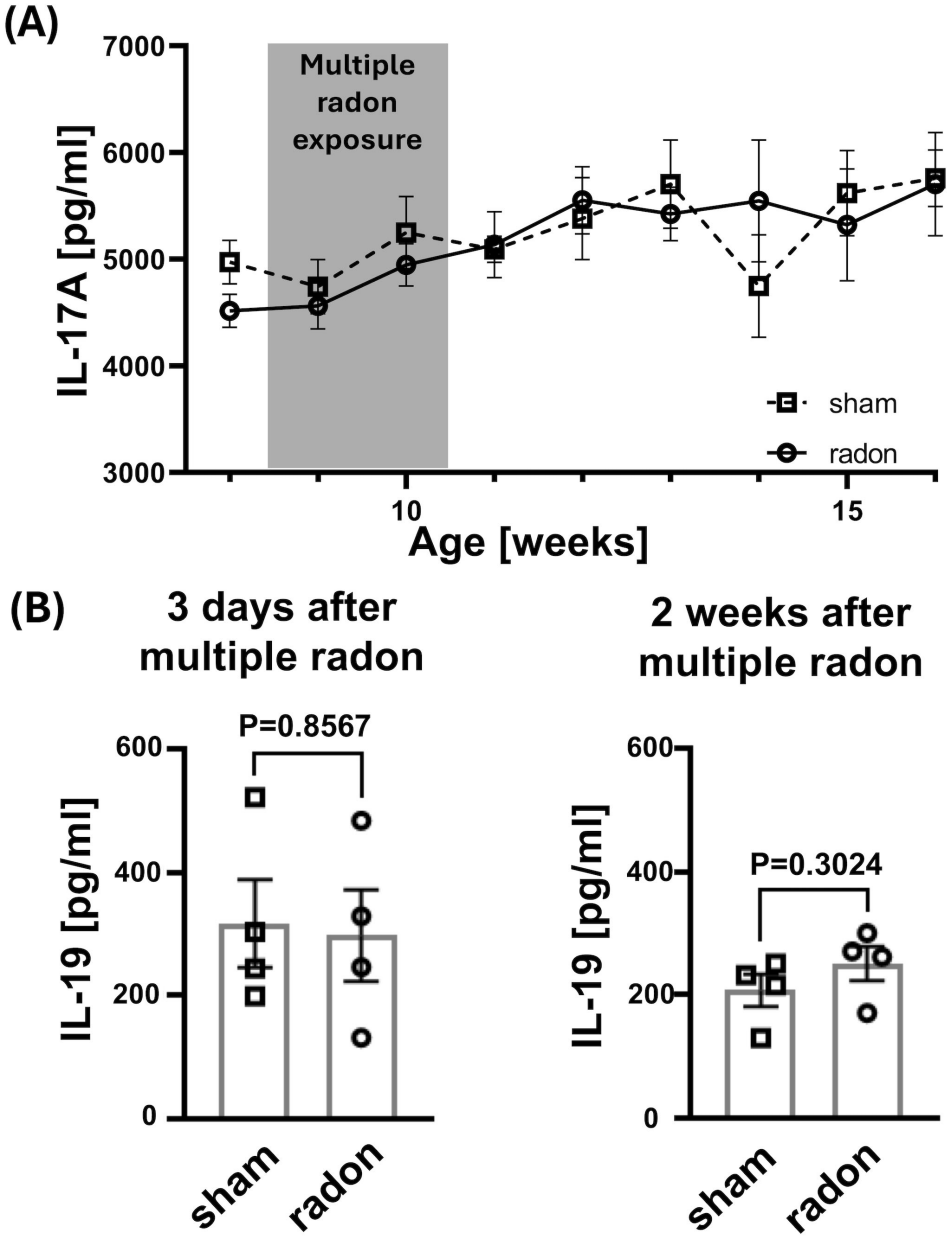
Serum concentrations of IL-17A and IL-19 in CD11c-IL-17A^ind/ind^ mice is not affected after multiple radon exposures. Cytokine concentrations in serum of CD11c-IL-17A^ind/ind^ mice were measured at different time points before, during, and after 10 exposures to a radon activity concentration of 39.2 ± 2.0 kBq/m³ or sham treatments (1h each) using uncoated ELISA kits from Invitrogen AG. **(A)** Serum concentrations of IL-17A over a period of 9 weeks; unpaired *t*-test for each time point since the Shapiro–Wilk test indicated normal distribution for the datasets, *n* (sham) = 3–13, *n* (radon) = 4–17; mean values ± SEM. **(B)** Serum concentrations of IL-19, 3 days and 2 weeks after the end of the treatment period; unpaired *t*-test since normal distribution can be assumed due to the Shapiro–Wilk test; *n* (sham, radon) = 4 for both time points; mean values ± SEM.

The serum concentration of IL-17A was measured before and up to 8 weeks after starting the multiple radon exposures. No radon-induced changes were observed compared to controls. Furthermore, no changes in IL-17A expression were detected over time (**Figure 2A**).

The serum concentration of IL-19 was analyzed 3 days and 2 weeks after the last treatment session as the most pronounced difference in the score of skin reactions was detectable during and up to 3 weeks after multiple radon exposures. Consistently with IL-17A, the serum concentration of IL-19 did not differ between radon-exposed and sham-treated mice (**Figure 2B**).

These data indicate that transgenic overexpression of IL-17A in CD11c-IL-17A^ind/ind^ mice does not change after radon exposures. This also accounts for its downstream amplifier IL-19.

### 3.3 Depletion of pDC in lymph nodes but not in the spleen of CD11c-IL-17A^ind/ind^ mice after multiple radon exposures

The onset of psoriatic skin inflammation is promoted by different DC subtypes, with pDCs being the initiators of the inflammatory cascade and cDCs being the activators for Th1 and Th17 cells. The resulting imbalance of regulatory T (Treg) and effector T cells including the associated cytokine profile further promotes the inflammatory response (11, 16).

**Figure 3:**
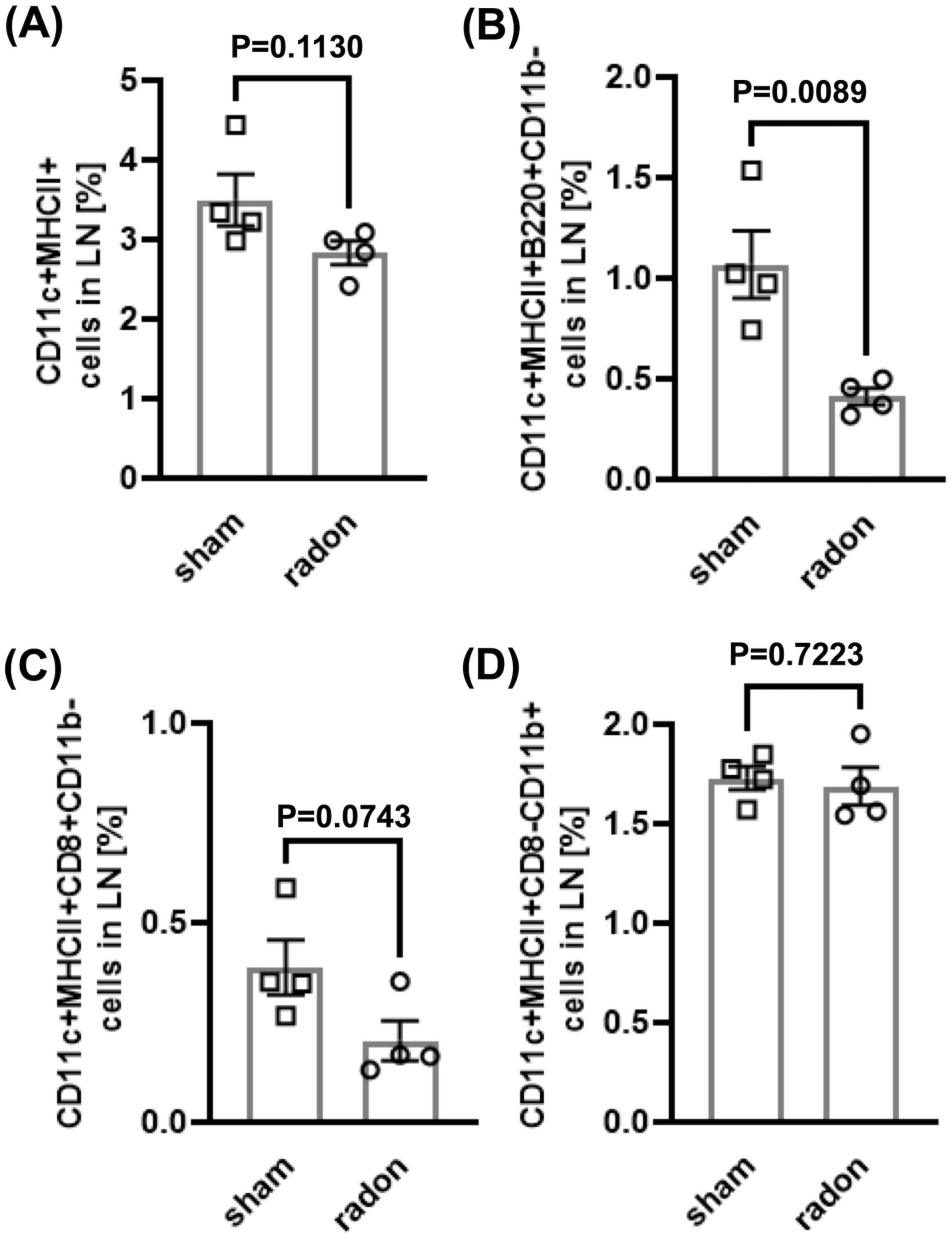
Multiple radon exposures reduce the amount of pDC in lymph nodes of CD11c-IL17A^ind/ind^ mice. Flow cytometric analyses of cervical lymph nodes of CD11c-IL17A^ind/ind^ mice to quantify different DC subtypes 3 days after 10 exposures to a radon activity concentration of 39.2 ± 2.0 kBq/m³ or sham treatment for 1h each. Gating strategies are shown in **Supplementary Figure S3**. **(A–D)** Percentage of DC subtypes in lymph nodes: **(A)** Mature DC: CD11c^+^MHCII^+^; **(B)** pDC: CD11c^+^MHCII^+^B220^+^CD11b^−^, **(C)** cDC1: CD11c^+^MHCII^+^CD8a^+^CD11b^−^; **(D)** cDC2: CD11c^+^MHCII^+^CD8a^−^CD11b^+^. Mann–Whitney *U* test when Shapiro–Wilk indicated no normal distribution of data, unpaired *t*-test when the Shapiro–Wilk test showed normal distribution; *n* (sham, radon) = 4; mean values ± SEM. All indicated values are related to the total number of singlets in lymph nodes or spleen. cDC, classical dendritic cells; DC, dendritic cell; LN, lymph node; pDC, plasmacytoid dendritic cell.

To investigate if multiple radon exposures can induce systemic changes in the inflammatory status of DCs and T cells, we conducted immunophenotyping in cervical LNs and spleen of radon- and sham-treated CD11c-IL-17A^ind/ind^ mice 3 days and 2 weeks after the last session. In LNs, we detected a decrease in the percentage of mature DCs (CD11c^+^MHCII^+^), pDCs (CD11c^+^MHCII^+^B220^+^CD11b^−^), and cDC1 (CD11c^+^MHCII^+^CD8a^+^CD11b^−^) 3 days after radon exposures as compared to sham-treated mice, but only with a statistical significance for pDCs (**Figures 3A–C**). No differences were found in cDC2 (CD11c^+^MHCII^+^CD8a^−^CD11b^+^) (**Figure 3D**), migratory DCs (CD11c^int^MHCII^high^), and LN-resident DCs (CD11c^high^MHCII^int^) at this early time point (**Table 1**; **Supplementary Figures S3C, D**). Additionally, the fractions of Th cells (CD3e^+^CD4^+^), cytotoxic T (Tc) cells (CD3e^+^CD4^−^), Treg cells (CD3e^+^CD4^+^CD25^+^), and Th17 cells (CD3e^+^CD4^+^CCR6^+^) showed no differences in LN of radon- and sham-treated animals at the 3-day time point (**Table 1**, **Supplementary Figures S3I–L**). Two weeks after the last radon exposure and in line with a transient effect, the identified effects in the immune cell status, namely, for DC, were no longer detectable in LN (**Supplementary Figures S4A–D, I–L**).

**Table 1:** Additional to **Figure 3**, results of flow cytometric analyses of cervical lymph nodes and spleen of CD11c-IL17A^ind/ind^ mice 3 days after multiple radon exposures or sham treatment to quantify different DC and T-cell subtypes.

Cell type and organ	Mean value ± SEM Sham (*n*= 4)	Mean value ± SEM Radon (*n* =4)	*P*-value (Mann–Whitney *U* or *t*-test) Radon vs. sham
CD11c^+^MHCII^+^ Mature DC in LN	3.495 ± 0.324	2.835 ± 0.148	0.1130
CD11c^+^MHCII^+^B220^+^CD11b^−^ pDC in LN	1.068 ± 0.168	0.411 ± 0.041	0.0089
CD11c^+^MHCII^+^CD8a^+^CD11b^−^ cDC1 in LN	0.388 ± 0.069	0.204 ± 0.050	0.0743
CD11c^+^MHCII^+^CD8a^−^CD11b^+^ cDC2 in LN	1.729 ± 0.058	1.688 ± 0.094	0.7233
CD11c^int^MHCII^high^ Migratory DC in LN	2.11 ± 0.255	1.458 ± 0.338	0.1895
CD11c^high^MHCII^int^ Resident DC in LN	3.160 ± 0.197	2.498 ± 0.303	0.1855
CD11c^+^MHCII^+^ Mature DC in spleen	4.395 ± 0.149	3.835 ± 0.791	0.5124
CD11c^+^MHCII^+^B220^+^CD11b^−^ pDC in spleen	1.795 ± 0.130	1.484 ± 0.453	0.5339
CD11c^+^MHCII^+^CD8a^+^CD11b^−^ cDC1 in spleen	0.540 ± 0.099	0.573 ± 0.250	0.9068
CD11c^+^MHCII^+^CD8a^−^CD11b^+^ cDC2 in spleen	1.146 ± 0.160	1.035 ± 0.129	0.6078
CD3e^+^CD4^+^ Th cells in LN	33.450 ± 1.548	32.375 ± 0.386	0.5256
CD3e^+^CD4^−^ Tc cells in LN	28.450 ± 1.477	27.100 ± 1.801	0.5834
CD3e^+^CD4^+^CD25^+^ Treg cells in LN	4.508 ± 0.378	4.324 ± 0.319	0.7229
CD3e^+^CD4^+^CCR6^+^ Th17 cells in LN	0.938 ± 0.086	0.827 ± 0.093	0.4149
CD3e^+^CD4^+^ Th cells in spleen	15.353 ± 0.886	13.050 ± 0.801	0.1021
CD3e^+^CD4^−^ Tc cells in spleen	11.65 ± 0.359	10.96 ± 0.370	0.2298
CD3e^+^CD4^+^CD25^+^ Treg cells in spleen	2.383 ± 0.157	2.129 ± 0.146	0.2816
CD3e^+^CD4^+^CCR6^+^ Th17 cells in spleen	0.515 ± 0.064	0.441 ± 0.060	0.4301

For gating strategies, see **Supplementary Figure S3**; cDC, classical dendritic cells; DC, dendritic cell; LN, lymph node; pDC, plasmacytoid dendritic cell; Tc, cytotoxic T cell; Th, T helper cell; Treg, regulatory T cell.

In contrast, in spleen, no differences were detected in radon- and sham-treated mice in the concentrations of the investigated DC and T-cell subtypes, neither for the 3-day (**Table 1**; **Supplementary Figures S3E–H, M–P**) nor for the 2-week time point (**Supplementary Figures S4E–H, M–P**). Taken together, the results show a radon-induced reduction of pDC in LNs but not in spleen of CD11c-IL-17A^ind/ind^ mice after multiple radon exposures.

### 3.4 No significant alteration of the immune status in the skin of CD11c-IL-17A^ind/ind^ mice after multiple radon exposures

As shown above, multiple radon exposures significantly delayed the development of severe skin lesions in CD11c-IL-17A^ind/ind^ mice. We hypothesized that this observation is related to the immune status in the skin. Therefore, we performed an immunohistological analysis with skin samples. Only a small percentage of mice showed psoriatic plaques at the time points of analysis. Thus, mainly non-lesional skin samples were analyzed to generate comparable datasets of radon- and sham-exposed mice.

**Figure 4:**
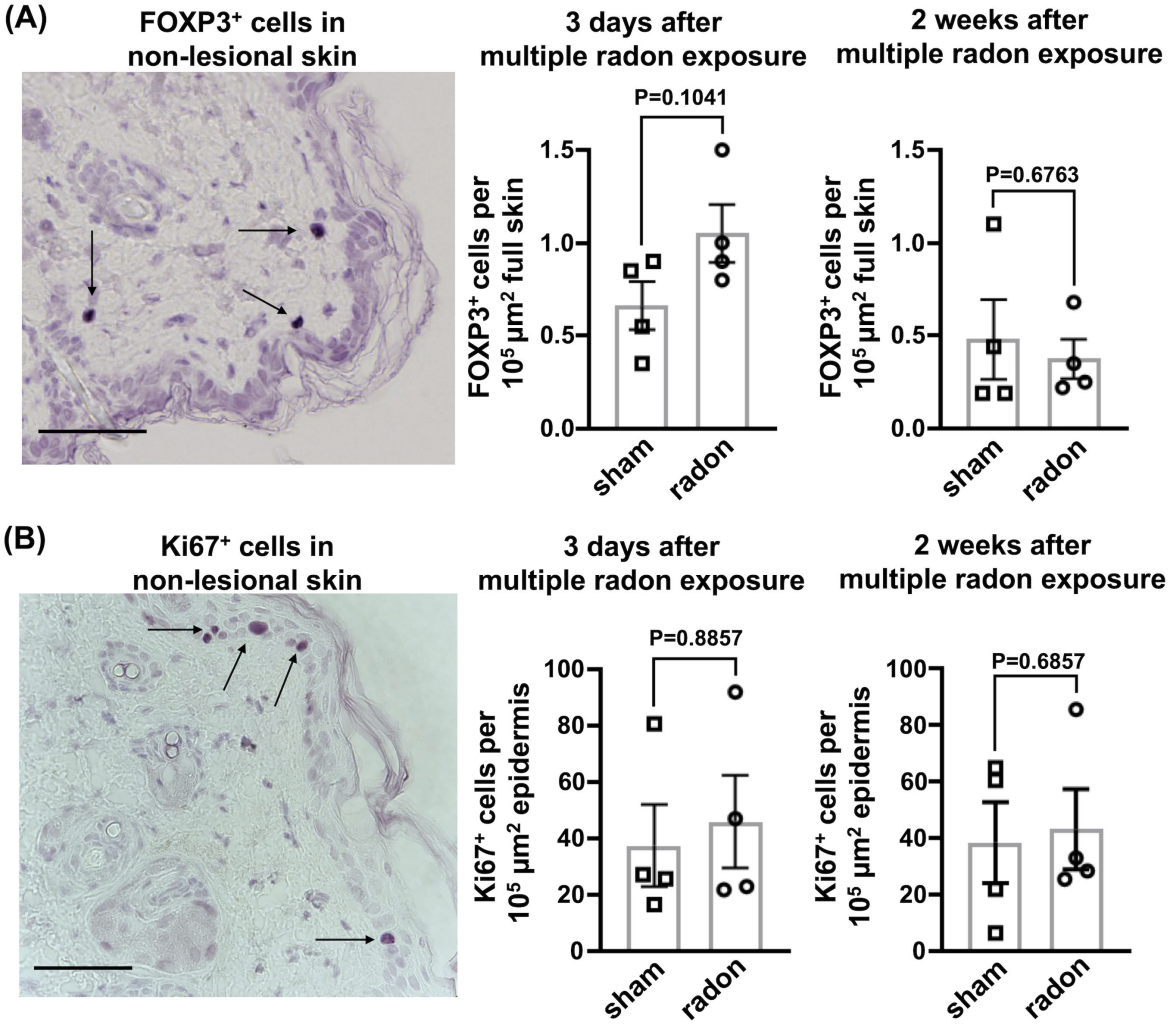
In the non-lesional back skin of CD11c-IL17A^ind/ind^ mice, multiple radon exposures show no significant effects on FOXP3^+^ and Ki67^+^ cells. Immunohistochemical staining to analyze protein production in the non-lesional back skin of CD11c-IL17A^ind/ind^ mice 3 days and 2 weeks after 10 consecutive exposures to a radon activity concentration of 39.2 ± 2.0 kBq/m³ for 1h each as compared to sham treatment. **(A)** Representative immunostaining of FOXP3 (arrow) in non-lesional back skin, indicating the presence of Treg cells and the amount of FOXP3^+^ cells 3 days or 2 weeks after the exposure period; cell numbers normalized to 10^5^ µm^2^ full skin; scale bar = 50 µm; unpaired *t*-test since normal distribution can be assumed due to the Shapiro–Wilk test; *n* (sham, radon) = 4; mean values ± SEM. **(B)** Representative immunostaining of Ki67 (arrow) in non-lesional back skin, indicating proliferative keratinocytes and the amount of Ki67^+^ cells 3 days or 2 weeks after multiple radon exposures; cell numbers normalized to 10^5^ µm^2^ epidermis; scale bar = 50 µm; Mann–Whitney *U* test since the Shapiro–Wilk test showed no normal distribution; *n* (sham, radon) = 4; mean values ± SEM.

To investigate if radon exposure leads to local immunosuppressive effects in the skin, we stained for FOXP3, a transcription factor specific for Treg cells that counteract psoriasiform inflammation (24). Additionally, we stained for the proliferation marker Ki67 to analyze if the hyperproliferation of keratinocytes, which is one hallmark of psoriasis (17, 25), is affected by radon treatment. Neither the fraction of FOXP3^+^ cells nor Ki67^+^ proliferating cells detected 3 days and 2 weeks after the last of multiple radon exposures were significantly different from controls (**Figure 4**).

We aimed at identifying neutrophils (via Ly6G staining), accumulating in psoriatic skin of CD11c-IL-17A^ind/ind^ mice (19), and Th17 cells (via RORγT staining), releasing IL-17A, both key events in the pathomechanism of human plaque psoriasis (13). As expected, in the lesional back skin of CD11c-IL-17A^ind/ind^ mice, both markers were detected (**Supplementary Figure S5**), but not in non-lesional skin. As only a few plaques were present at this early time point, statistical analysis was not possible. However, if a plaque could be analyzed, the number of neutrophils and Th17 cells in lesional skin was comparable in radon-exposed and sham-treated mice. Taken together, Th17 cells and neutrophils, two key immune cell types in psoriatic lesions, as well as keratinocyte proliferation in the skin of CD11c-IL-17A^ind/ind^ mice were not affected after radon exposures.

### 3.5 Transcriptome changes in non-lesional skin indicate an impact of multiple radon exposures on the initial phase of plaque formation

As flow cytometric and histological analysis did not reveal a clear immune cell signature supporting the significant clinical improvement in skin reactions of CD11c-IL-17A^ind/ind^ mice after multiple radon exposures, we next used an unbiased approach. We performed a comprehensive transcriptome profiling by RNA sequencing of non-lesional skin samples to quantify gene expression in radon-exposed CD11c-IL-17A^ind/ind^ mice and sham-treated animals.

**Figure 5:**
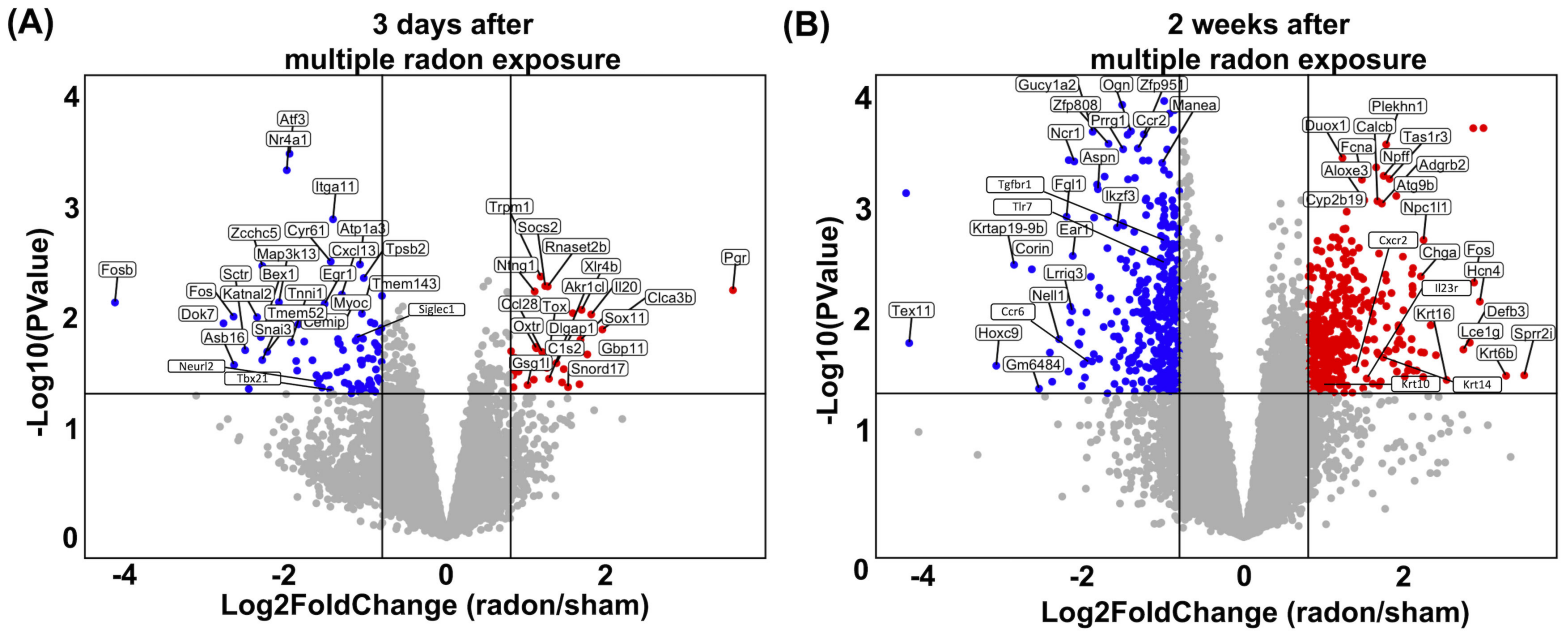
The transcription of numerous genes is affected in the non-lesional back skin of CD11c-IL17A^ind/ind^ mice after multiple radon exposures. Comprehensive transcriptome profiling shows a differential expression in numerous genes that evolves over time in the non-lesional back skin of CD11c-IL17A^ind/ind^ mice after radon exposures. Transcriptome profiling to identify most differentially expressed genes in the non-lesional back skin of CD11c-IL17A^ind/ind^ mice 3 days or 2 weeks after 10 consecutive exposures to a radon activity concentration of 39.2 ± 2.0 kBq/m³ for 1h each as compared to sham treatment. **(A, B)** Volcano plots indicate the relative difference of expression level between radon- and sham-exposed mice for each gene at the **(A)** 3-day time point and **(B)** 2-week time point; red: increased expression level of a gene after radon exposure as compared to sham treatment; blue: decreased gene expression level after radon exposure as compared to sham treatment; gray: no difference in gene expression level in radon- and sham-exposed mice.

An overview of the significant changes in the entirety of the analyzed genes is depicted in volcano plots for 3 days and 2 weeks after radon treatment. As shown in **Figures 5A, B**, radon exposure affected the transcription of numerous genes even in non-lesional skin. These changes were very different for the time points investigated, i.e., 3 days (**Figure 5A**) and 2 weeks (**Figure 5B**) after treatment, pointing to transient effects that evolved over time after the end of multiple radon exposures.

Additionally, we interrogated differences in the expression levels of distinct genes that are related to psoriasis and radiation response.

**Figure 6:**
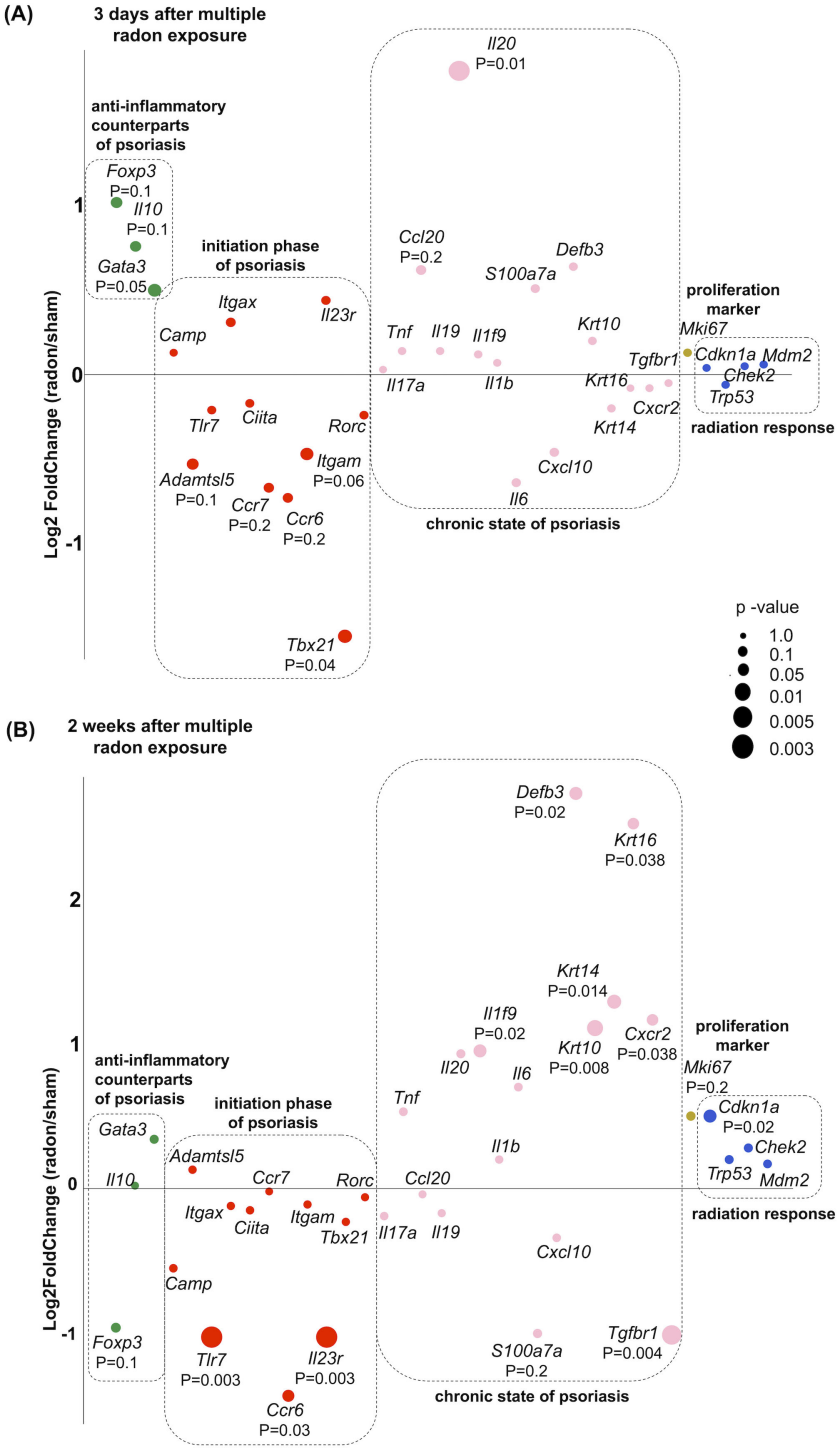
Early time point after multiple radon exposures; the non-lesional back skin of CD11c-IL17A^ind/ind^ mice shows effects in genes that are anti-inflammatory or related to the initiation phase of psoriasis, both potentially counteracting the disease. Transcriptome profiling to analyze gene expression in the non-lesional back skin of CD11c-IL17A^ind/ind^ mice 3 days or 2 weeks after 10 consecutive exposures to a radon activity concentration of 39.2 ± 2.0 kBq/m³ for 1h each as compared to sham treatment. Dot plots showing relative differences between radon- and sham-exposed mice in the expression level of selected psoriasis relevant genes **(A)** 3 days and **(B)** 2 weeks after treatment. Genes related to anti-inflammatory counterparts of psoriatic reaction (green), initiation phase (red) and chronic state of psoriasis (rose), proliferation of keratinocytes (yellow ocher), and response to ionizing radiation (blue); *n* (sham, radon) = 4.

The expression of the transcription factor *Gata3*, specific for T cells counteracting the psoriatic inflammation by association with Th2 cells (26), was upregulated 3 days after radon exposures (**Figure 6A**), while this was not detectable anymore at 2 weeks after exposures (**Figure 6B**). The expression of the anti-inflammatory factors *Il10* and *Foxp3* was not modified.

Regarding the initiation phase of psoriasis, the expression of the transcription factor *Tbx21* associated with Th1 cells was significantly downregulated at 3 days after exposure (27) (**Figure 6A**), while this was no longer detectable at the later time point (2 weeks, **Figure 6B**).

Not at 3 days, but at 2 weeks after radon exposure, a significant downregulation of the genes *Tlr7*, *Ccr6*, and *Il23r* was detected. These genes encode for relevant receptors involved in the initiation of the immune response promoting the progression of psoriasis (28–31) (**Figure 6B**).

Genes encoding autoantigens involved in psoriasis and related to the initiation phase were not significantly changed, i.e., *Adamtsl5*, encoding a psoriasis-related melanocyte-derived autoantigen (15), and *Camp*, encoding the autoantigen LL-37 (14). Also not affected by radon exposure were genes involved in the immune response, *Itgax* (T-cell marker, elevated in psoriasis), *Ciita* (marker for DC), and *Rorc* (marker for Th17 cells) (32–34).

While we have observed attenuated development of skin lesions in CD11c-IL-17A^ind/ind^ mice after radon exposures, the gene expression of soluble inflammatory or differentiation factors produced by immune cells or keratinocytes that are related to the chronic state of psoriasis was either not affected or significantly increased as in the case of *Il20* at the early time point or of *Il1f9* and *Defb3* at the late time point (35, 36). One exception at the late time point was *Tgfbr1*, part of the disease promoting signaling in psoriasis, which was significantly downregulated (37).

Additionally, genes that encode keratins 10, 14, and 16 were significantly upregulated at the 2-week time point, indicating a complex relation to keratinocyte differentiation (38). Furthermore, the proliferation marker *Mki67* as well as typical radiation-induced genes, i.e., *Trp53, Chek2*, and *Mdm2* (39, 40), were not differentially expressed after radon exposures, except *Cdkn1a* (target gene of *Trp53*) that was significantly upregulated 2 weeks after exposure.

Since the identified differences in the expression levels of distinct psoriasis-related genes indicated transient immunosuppressive effects after multiple radon exposures in CD11c-IL-17A^ind/ind^ mice, we performed a pathway enrichment analysis as described in detail in the **Supplementary Materials** (**Supplementary Figures S6**, **S7**). This revealed that radon exposure mainly affects the organization and maturation of different skin compartments, ion transport and regulation, kinases and T cells, biosynthesis, as well as processes associated with ultraviolet (UV) response. In conclusion, the transcriptome profiling in the skin of CD11c-IL-17A^ind/ind^ mice revealed a transient upregulation of the expression of psoriasis-suppressive genes, whereas genes related to the initiation of psoriasis were downregulated after multiple radon exposures. Genes related to keratinocyte proliferation were not differentially expressed after radon treatment or showed a complex pattern for keratinocyte differentiation, and the cell cycle inhibitor Cdkn1a was significantly upregulated.

### 3.6 Radon and UV radiation induced partially overlapping transcriptional changes in the skin

Radon exposure is a radiation-based and non-invasive treatment, similar to UV irradiation, which is one of the conventional therapy options for psoriatic skin lesions. As described in more detail in the **Supplementary Materials**, we compared the transcriptome profile of non-lesional skin from CD11c-IL-17A^ind/ind^ mice after radon exposure with three GEO-available gene expression analyses of human psoriatic skin lesions after UV treatment to assess the relevance of our results for patients. This comparison revealed 33 differentially expressed genes (DEGs) (*P*<0.05) that are identical after radon and UV treatment (**Supplementary Figure S8**). According to UniProt and GeneCards, the DEGs that are identical after radon and UV therapy are involved in the regulation of cell proliferation and differentiation, calcium concentration, T-cell activation, innate immunity, class I MHC antigen presentation, and TNF-alpha signaling. In addition, these DEGs modulate the MAPK cascade and ERK signaling, with ERK1/2 being of high interest in the pathogenesis of psoriasis because it serves as a target for glucocorticoids, which are used as a standard treatment (41, 42). In summary, this comparative gene expression analysis of radon- and UV-exposed psoriasiform skin lesions shows partially overlapping changes in psoriasis-relevant pathways.

## 4 Discussion

Although psoriasis is listed as an indication for radon therapy, reliable patient studies and preclinical data supporting its effectiveness are lacking. Here, we demonstrate for the first time that radon treatment can significantly delay plaque formation in a psoriatic mouse model where placebo effects can be excluded. Thus far, in line with all treatments used against psoriasis, the effect elicited by radon exposure that we observed is temporary, implying the necessity to repeat the treatment at regular annual intervals (43).

Other treatment options for psoriasis have been improved, but resistances may develop (e.g., using antibodies—so-called “biologicals”—against essential components of the pathomechanism of psoriasis), or side effects of varying severity may occur using classical systemic medications (e.g., methothrexate and ciclosporin) (43).

In CD11c-IL-17A^ind/ind^ mice, CD11c-Cre-mediated removal of a *loxP*-flanked transcriptional STOP cassette drives transgenic IL-17A expression through a constitutively active promoter, which is not expected to be regulated by radon exposure (19, 44). This model-specific feature is confirmed by unchanged serum concentration of IL-17A and downstream IL-19 after radon treatment. In this context, it is remarkable that the development of skin lesions is significantly delayed and less severe, highlighting the efficacy of radon treatment. Moreover, the unaltered IL-17A expression could explain why the mitigating effect is only transient and lesion development and severity are equivalent to those of sham-treated animals 2–4 weeks after the end of radon exposure. Of note, this transient nature of the beneficial effects in mice is consistent with patient reports of a reduction of psoriasis plaques that persisted for approximately 6 months after radon inhalation therapy. Taken together, this strongly suggests that radon exposure generally leads to a temporary relief of psoriasis symptoms.

On the other hand, long-term exposure to environmental background concentrations of radon is the second-leading cause of lung cancer (45). Epidemiologic studies estimating an increased risk of lung cancer from temporary radon inhalation therapy are not yet available. Studies in rats show an inverse dose–response effect, with longer exposures at lower dose rates causing more lung carcinomas than shorter exposures at higher dose rates (46). Therefore, the risk of developing radon-induced cancer and the benefit of a radon treatment of psoriasis symptoms must be carefully weighed on a case-by-case basis.

Immunophenotypic analysis of cervical LN shows a significant depletion of pDCs, which are one of the main early drivers of psoriatic skin inflammation (11, 16). The pDC depletion could be one main reason for the clinical improvement of plaque induction in CD11c-IL-17A^ind/ind^ mice. Transcriptome profiling of non-lesional skin also reveals that local expression of genes encoding initial psoriatic factors such as the Th1 cell marker *Tbx21* (25, 47) decreases shortly after radon exposure, while expression of a factor counteracting psoriatic development, i.e., Th2 (*Gata3*), increases. These changes may also contribute to the improvement of skin lesions in CD11c-IL-17A^ind/ind^ mice.

The question arises, which are the relevant target organs of radon exposure that lead to the significant delay in plaque formation and the underlying molecular and cellular effects? The incorporation of radon gas and its decay products into the body is complex, and thus, the identification of target organs and the determination of the applied dose must be carefully considered (3, 8). Radon gas intake occurs via the lungs or skin and subsequent diffusion. Most of the inhaled radon gas is rapidly exhaled and only ~1% is absorbed by the blood, from where it is distributed via the blood stream and accumulates in the organs depending on their blood perfusion rate and radon solubility (3, 8–10). A major part of the total dose is deposited during the decay of solid progeny present in the ambient air, which adhere to the surface of the skin and lung epithelium (3, 8).

As with the skin, direct irradiation during radon decay should be considered for LNs, because LNs are located in the subcutaneous fat, where radon has a 10-fold higher solubility than in other tissues (9, 48–51). Hence, radon is likely to accumulate in the cervical fat, where it decays and the emitted alpha-particles irradiate the LNs. This is consistent with the idea that a major reason for the clinical improvement of skin lesions in CD11c-IL-17A^ind/ind^ mice may be the observed reduction of pDCs in the LNs.

In general, the mitigating effects of radon exposures on psoriatic inflammation can be mediated via local factors in the skin and lung epithelium, systemically throughout the body, or by a combination of both. Our results demonstrate that in transgenic CD11c-IL-17A^ind/ind^ mice, a combination of decreased psoriasis initiation factors and reduced pDC numbers in LNs exerts a systemic effect leading to the improvement of skin lesions. Presumably, these effects are mediated secondarily after irradiation of individual (immune) cells, since typical radiation effects were only detected for the induction of *Cdkn1a*, not for *Trp53*, *Chek2*, and *Mdm2* (39, 40), which are not detectable in the skin after radon exposure.

Given the thickness of the epidermis of mice and humans (<25 and >50 µm, respectively) (52) and the range of alpha-particles (20–100 µm) (53), it is likely that deeper human skin layers are partially not irradiated. In this context, systemic rather than local effects in patients might become more relevant. Furthermore, the transgenic model-specific feature of permanent IL-17A production in CD11c-IL-17A^ind/ind^ mice must be taken into account. While IL-17A-producing cells and downstream factors cannot be influenced in these mice, these components could be targeted by radon in patients and promote the systemic therapeutic effect.

The relevance of our results for explaining the immunosuppressive effects of radon exposure becomes evident when compared to UV radiation therapy, a commonly applied immunoregulatory treatment of psoriatic skin lesions that, like radon, is non-invasive and radiation-based. In agreement with the known effects of UV therapy, reported for skin biopsies and blood of UV-treated patients (54–56), transcriptome profiling of radon-exposed, non-lesional mouse skin revealed a considerable number of DEGs, which are identical for UV and radon exposure (53–55). However, one limitation of our study is the relatively low number of animals per group, especially for the defined time points of 3 days and 2 weeks after multiple radon exposures (*n*=4). The analysis of a higher number of animals could enhance the robustness of the results, particularly with respect to the molecular analysis of the pathways.

In conclusion, we provide the first *in vivo* evidence for the mitigating effects of radon exposure in a preclinical mouse model with spontaneous and chronic features of psoriatic skin inflammation. Our results indicate that a combination of transient local and systemic effects mediates the delayed plaque formation following radon exposure in CD11c-IL-17A^ind/ind^ mice. Whether similar effects occur in patients with psoriasis after radon therapy should be investigated in a larger patient study, including a risk–benefit analysis estimating the cancer risk associated with temporary radon inhalation in galleries.

## Data Availability

The datasets presented in this study can be found in the NCBI GEO repository, accession GSE292505.
