# Supplementary material for: Radon exposure delays the development of skin lesions in a mouse model of psoriasis
Source: Front Immunol. 2025 Nov 18;16:1638483. doi: 10.3389/fimmu.2025.1638483 (PMC12669134; doi:10.3389/fimmu.2025.1638483)
Supplement: Supplementary file 1 [file DataSheet1.pdf]

Supplementary Material

1 Supplementary Methods

1.1 Scoring of skin lesions

Table 1: Severity score for skin reaction of CD11c-IL-17A $^{ind/ind}$ mice

Score	Skin reaction	Explanation
0	healthy skin	light skin, intact skin barrier
1	dry skin	rough skin
2	scaly skin	area with single scales
3	white plaque	defined thickened area covered with stacks of scales
4	single plaque with red surf	3 plus a little (bloody) scurf
5	>1 red plaque (4)	> 1 separate plaque with scurf
6	spread plaque	large area (≥ 0.5 x 0.5 cm on back; most part of ears) which is thickened and covered with scales & scurfs

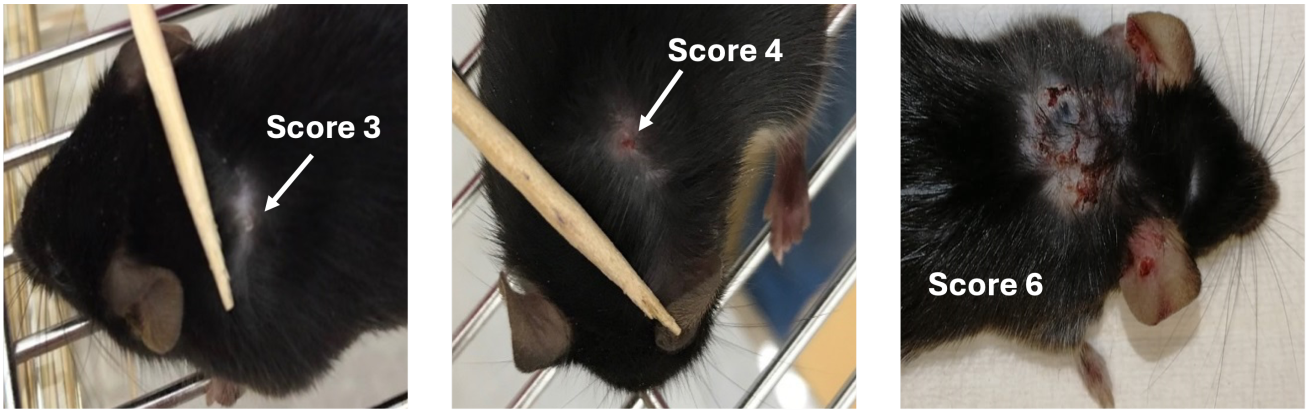

Supplementary Figure S1: Representative photos of CD11c-IL-17A$^{ind/ind}$ mice showing skin lesions related to score 3, 4 or 6 as described in Table 1.

1.2 Histology

Tissue samples were embedded in paraffin, cut (5 µm slices), deparaffinized and rehydrated. After antigen retrieval using citric acid buffer (pH6) or TRIS-EDTA buffer (pH9) skin sections were stained for Ki67 (monoclonal, SP6, abcam), FoxP3 (monoclonal, FJK 16s, eBioscience), RORγT (monoclonal, AFKJS-9, invitrogen) or Ly6g (monoclonal, EPR22909-135, abcam).

Proliferating cells in the skin, scored as Ki67$^+$ cells, were counted in 20 pictures out of 2 slices per sample (light microscope BX61); the area of epidermis was measured and Ki67$^+$ cells were normalized to an area of 10^5 µm^2. FOXP3$^+$ Treg cells were counted in scans of 2 complete slices per sample (slide scanner Axio Scan.Z1) and normalized to an area of 10^5 µm^2 of full skin.

1.3 Immunophenotyping of lymph nodes and spleen

Spleen and lymph nodes were disrupted mechanically using tissue strainers (40 or 100 µm; greiner bio-one). Red blood cells were lysed using a lysing buffer containing ammonium chloride, potassium hydrogen carbonate and EDTA. After the cell suspension was washed, centrifuged and resuspended, viable cells were counted using trypan blue staining and an automated cell counter (TC20™, Bio-Rad Laboratories). For staining, $2*10^6$ cells per sample were used. Before labeling with surface antibodies, cells were incubated in a blocking buffer containing Fc-Block (BD Bioscience). Antibody staining and flow cytometry analysis on a FACS Canto II (BD Bioscience) was performed in order to obtain DC and T cell distribution.

1.4 Gene set enrichment analysis after transcriptome profiling

Gene set enrichment analysis (GSEA) was performed in preranked mode with log2-ratios generated in DESeq2 differential gene expression analysis. Enrichment was tested for gene sets of the MSigDB GO-BP, Reactome and Hallmark pathways. Enrichments with a p-value of smaller than 0.05 and an FDR smaller than 25% were considered statistically significant. Cytoscape was used to visualize the results of pathway enrichment analysis.

2 Supplementary Results

2.1 Severity of skin lesions

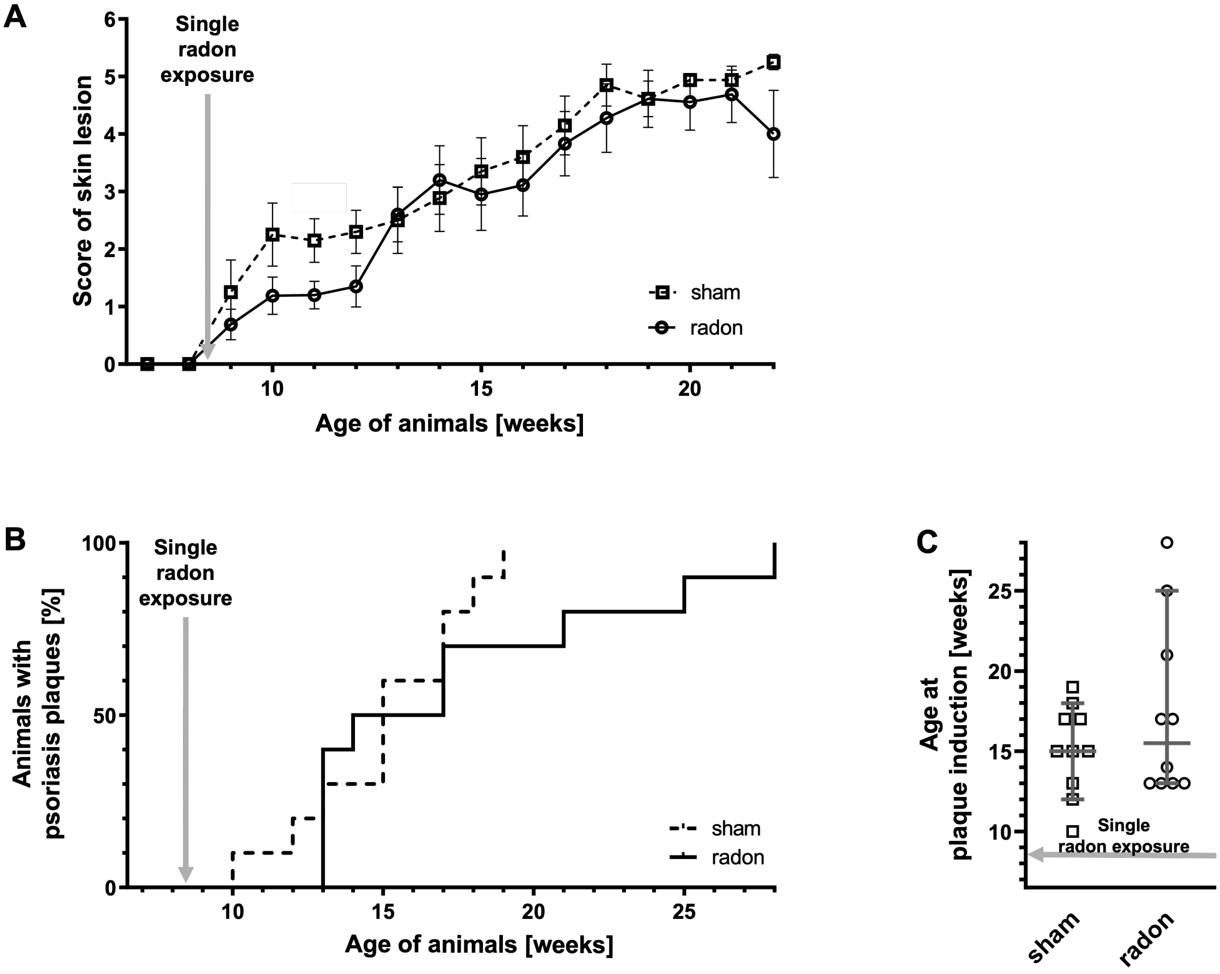

Supplementary Figure S2: Single radon exposure leads to a slightly delayed onset of plaque induction in CD11c-IL-17A$^{ind/ind}$ mice. 8.5-week-old (±3 days) CD11c-IL-17A$^{ind/ind}$ mice received a single radon exposure with an activity concentration of 539.0 ± 2.8 kBq/m³ or a sham treatment in our radon chamber for 1 hour. According to the score of 0 – 6 (Supplementary Table 1), the severity of skin reactions was examined 3 times per week; reaching score of 4 = time point of plaque induction. (A) Averaged score values of skin reactions over time for different ages in weeks; Mann-Whitney-U test, n(sham) = 8-10, n(radon) = 5-10; mean values ± SEM. (B) Kaplan-Meier-Plot with percentage of animals showing characteristic psoriasis plaques (score ≥ 4) at different ages in weeks; Log-rank (Mantel-Cox) test, n(sham, radon) = 10. (C) Age in weeks at plaque induction (reaching score ≥ 4); Mann-Whitney-U test, n (sham, radon) = 10; median with 95% confidence interval.

2.2 Analysis of DC and T cell subtypes in lymph nodes and spleen of CD11c-IL-17A$^{ind/ind}$ mice 3 days and 2 weeks after radon exposure

For the early analysis time point (3 days after multiple radon exposures), most of the investigated immune cell types in lymph nodes and spleen did not show significant differences between control and radon exposed mice. The respective plots of the results are shown in Supplementary Figure S3, including the gating strategy. The same data sets are presented in tabular form in the manuscript (Table 1).

In contrast to the early analysis time point, in lymph nodes only a trend for a reduction of pDC was recognizable at the 2-week time point. The other analyzed DC subtypes and T cell populations showed no change after radon exposure as compared to sham treatment at this time point (Supplementary Figure S4).

In spleen, just as at the early time point, no change in the DC and T cell subtypes was recognizable 2 weeks after multiple radon exposures (Supplementary Figure S4).

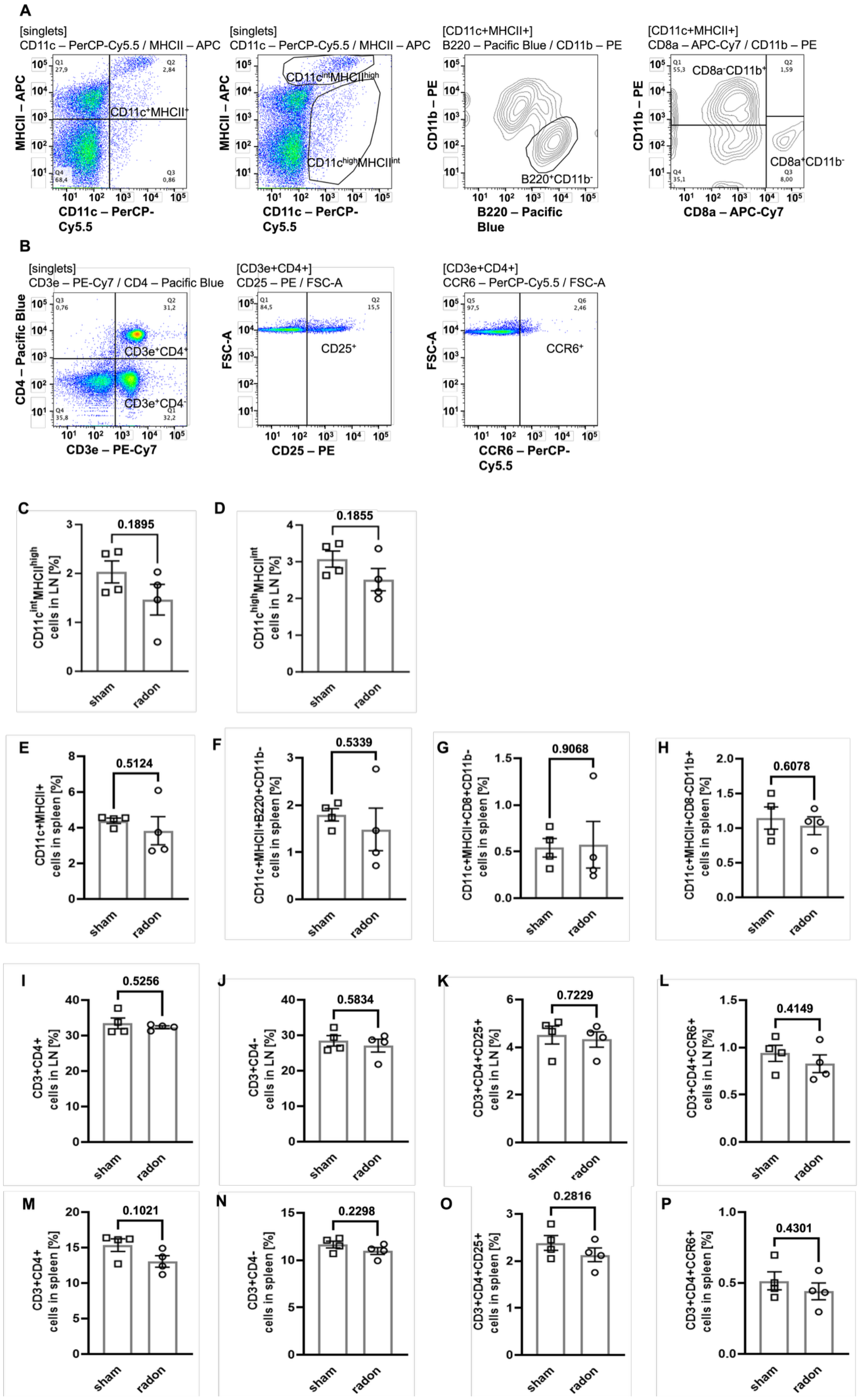

Supplementary Figure S3: No changes were detected in the proinflammatory status of some DC and T cell subtypes in lymph nodes and spleen of CD11c-IL17A$^{ind/ind}$ mice 3 days after multiple radon exposures. Flow cytometric analyses of cervical lymph nodes and spleen of CD11c-IL17A$^{ind/ind}$ mice to quantify different DC and T cell subtypes 3 days after 10 exposures to a radon activity concentration of 39.2 ± 2.0 kBq/m³ or sham treatments for 1 hour each. (A) Representative gating strategy for different DC subtypes: Cells were pre-gated in FSC-A / FSC-H and FSC-A / SSC-A to exclude

doublets and debris; afterwards, singlets were gated for different subtypes of DCs: Mature DC: $CD11c^{+}MHCII^{+}$; migratory DC: $CD11c^{int}MHCII^{high}$; resident DC: $CD11c^{high}MHCII^{int}$; pDC: $CD11c^{+}MHCII^{+}B220^{+}CD11b^{-}$; cDC1: $CD11c^{+}MHCII^{+}CD8a^{+}CD11b^{-}$; cDC2: $CD11c^{+}$ $MHCII^{+}CD8a^{-}$ $CD11b^{+}$. (B) Representative gating for different T cell subtypes: Again, to exclude doublets and debris cells were pre-gated in FSC-A / FSC-H and FSC-A / SSC-A; afterwards, gating for different T cell subtypes: Th: $CD3e^{+}CD4^{+}$; Tc: $CD3e^{+}CD4^{-}$; Treg: $CD3e^{+}CD4^{+}CD25^{+}$, Th17: $CD3e^{+}CD4^{+}CCR6^{+}$. (C-D) Percentage of different subtypes of DCs in lymph nodes. (E-H) Percentage of DC subtypes in spleen. (I-L) Percentage of T cell subtypes in lymph nodes. (M-P) Percentage of T cell subtypes in spleen. Mann-Whitney-U test when Shapiro-Wilk indicated no normal distribution of data, unpaired t test when Shapiro-Wilk test showed normal distribution; n(sham, radon) = 4 for lymph nodes and spleen; mean values ± SEM. All indicated values are related to the total number of singlets in lymph nodes or spleen. DC: dendritic cell, LN: lymph node, pDC: plasmacytoid DC, Tc: cytotoxic T cells; Th: T helper cell, Treg: regulatory T cell.

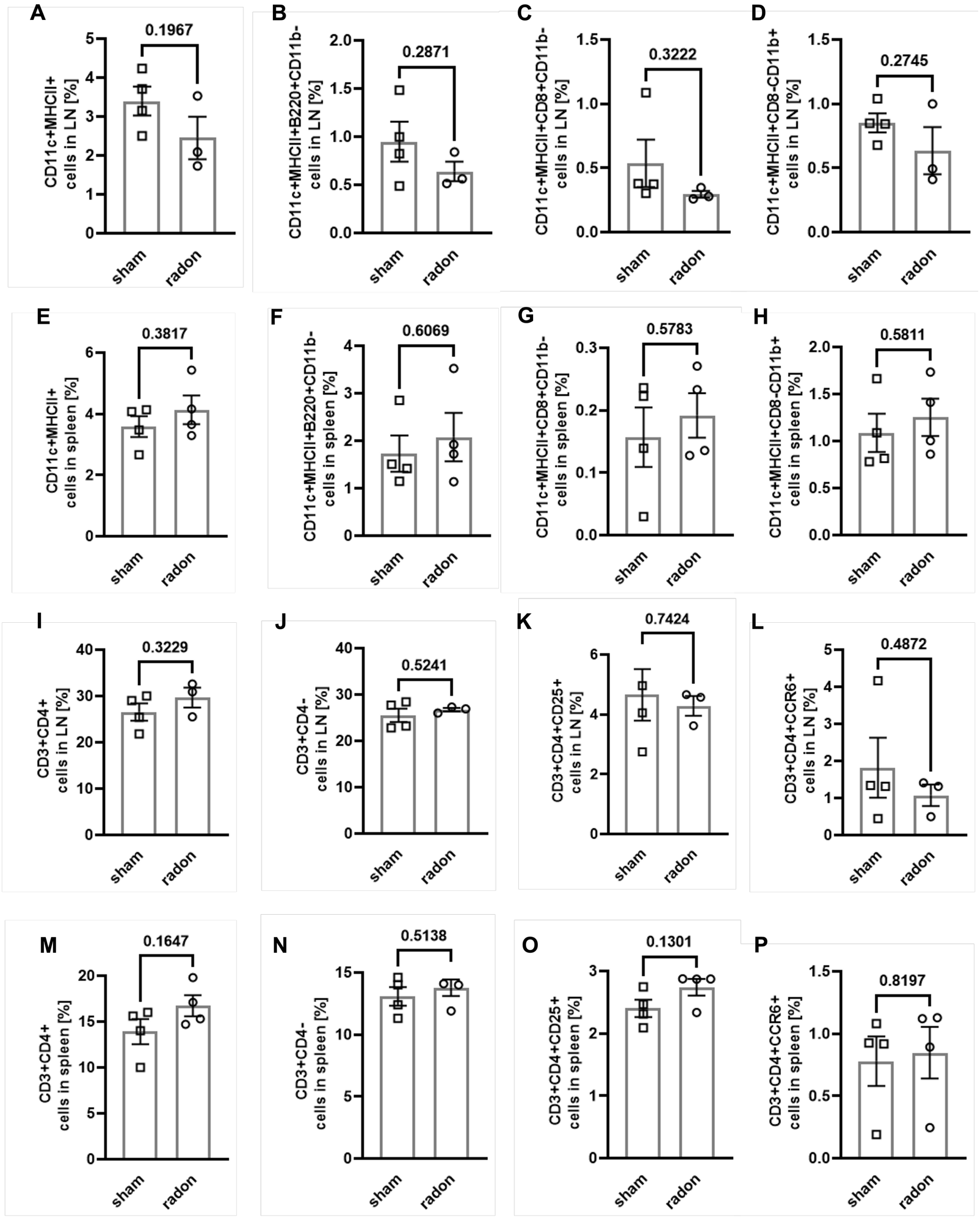

Figure S4: No changes were detected in the proinflammatory status of DC and T cell subtypes in lymph nodes and spleen of CD11c-IL17A$^{ind/ind}$ mice 2 weeks after multiple radon exposures. Flow cytometric analyses of cervical lymph nodes and spleen of CD11c-IL17A$^{ind/ind}$ mice to quantify different DC and T cell subtypes 2 weeks after 10 exposures to a radon activity concentration of 39.2 ± 2.0 kBq/m³ or sham treatments for 1 hour each. Gating strategies are shown in Supplementary Figure S3. (A-D) Percentage of different subtypes of DCs in lymph nodes. (E-H) Percentage of DC subtypes in spleen. (I-L) Percentage of T cell subtypes in lymph nodes. (M-P) Percentage of T cell subtypes in spleen. Mann-Whitney-U test when Shapiro-Wilk indicated no normal distribution of data,

unpaired t test when Shapiro-Wilk test showed normal distribution; n(sham, radon) = 3-4 for lymph nodes and spleen; mean values ± SEM. All indicated values are related to the total number of singlets in lymph nodes or spleen. DC: dendritic cell, LN: lymph node, pDC: plasmacytoid DC, Tc: cytotoxic T cells; Th: T helper cell, Treg: regulatory T cell.

2.3 Histological analysis of immune status in skin of CD11c-IL-17A$^{ind/ind}$ mice

To identify neutrophils and Th17 cells we stained for Ly6G and RorγT, respectively. Both markers were detected in lesional back skin of CD11c-IL-17A$^{ind/ind}$ mice (Supplementary Figure S5), but not in non-lesional skin. Due to the low number of animals showing psoriasis plaques at the time points of analysis a statistical analysis could not be performed. Anyway, we excluded differences in the amounts of Ly6G and RorγT in radon treated and sham exposed mice.

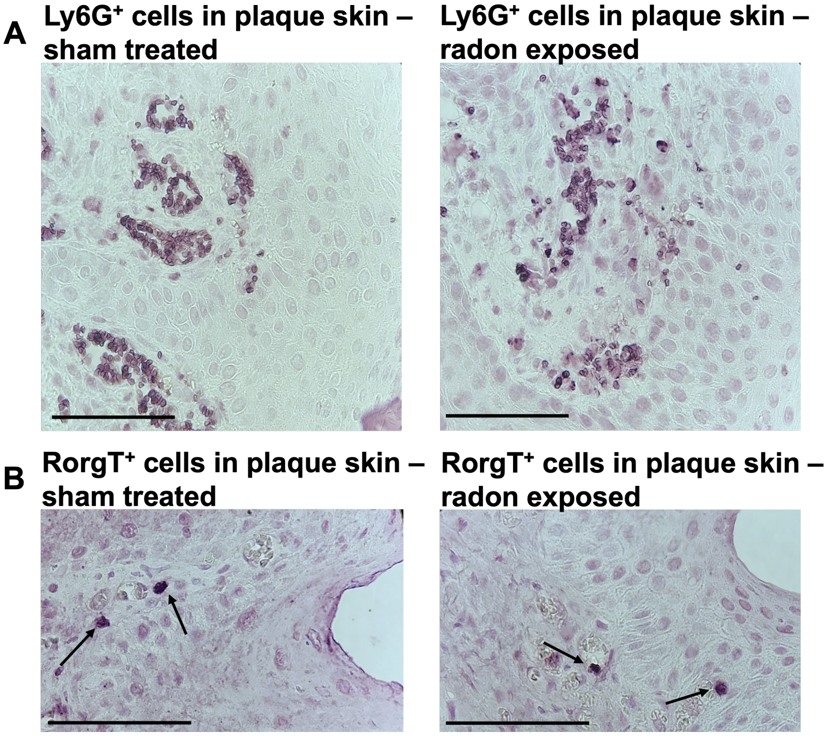

Supplementary Figure S5: In psoriatic plaques of CD11c-IL17A$^{ind/ind}$ mice, no effects could be recognized on protein levels of Ly6G and RorγT after multiple radon exposures. Immunohistochemical staining to analyze protein production in psoriatic plaques of CD11c-IL17A$^{ind/ind}$ mice 3 days after 10 consecutive exposures to a radon activity concentration of 39.2 ± 2.0 kBq/m^3 for 1 hour each. (A, B) Representative immunostaining of (A) Ly6g and (B) RORγT (arrow) in lesional back skin of sham and radon exposed mice; scale bar = 50 μm.

2.4 Pathway enrichment analysis based on transcriptome profiling in non-lesional skin of CD11c-IL-17A$^{ind/ind}$ mice and subsequent pathway enrichment analysis

To perform pathway enrichment analysis of DEGs early after radon treatment, we ranked the entire transcriptome data set of the 3-day time point regarding the extent of gene expression regulation in radon exposed mice as compared to sham treated animals. Afterwards, the ranking was used to perform gene set enrichment analysis (GSEA) of MSigDB Hallmark-, GO-biological process (BP) and Reactome-pathways. Using Cytoscape enrichment maps clusters of pathways were built according to gene set similarities representing higher-level pathway groups. For each pathway group, an averaged normalized enrichment score (NES) as well as the range of included NES was determined, as shown in Supplementary Figure S6. The enrichment maps can be found in Supplementary Figure S7.

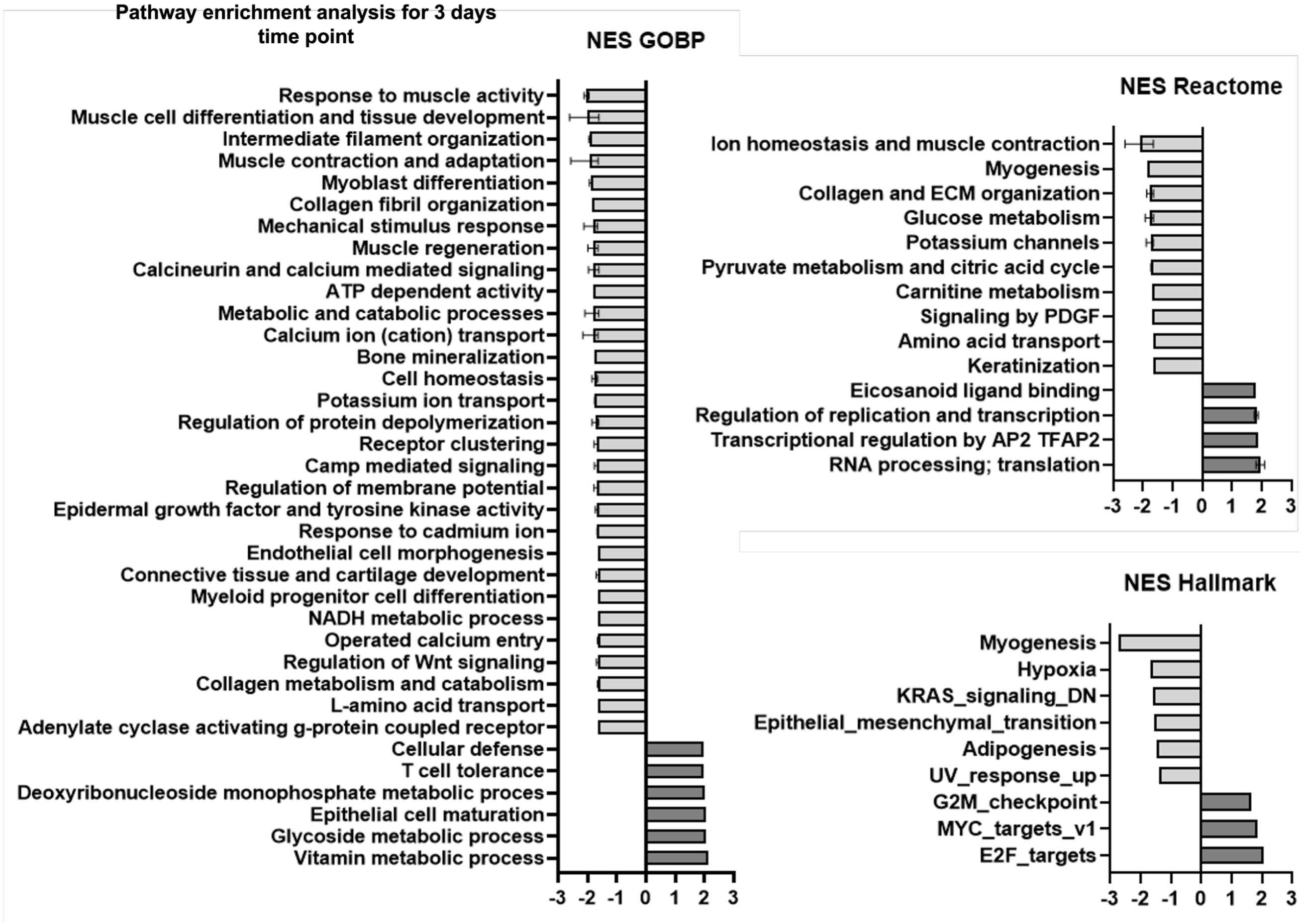

Supplementary Figure S6: Comprehensive transcriptome profiling shows that early regulated genes in non-lesional back skin of CD11c-IL17A$^{ind/ind}$ mice after radon exposures are involved in a variety of signaling pathways. Transcriptome profiling and subsequent pathway enrichment analysis to identify most differentially expressed genes and associated pathways in non-lesional back skin of CD11c-IL17A$^{ind/ind}$ mice 3 days after 10 consecutive exposures to a radon activity concentration of 39.2 ± 2.0 kBq/m³ for 1 hour each as compared to sham treatment. Averaged NES shows the enrichment status of higher-level pathway groups in which significant regulated genes after radon treatment are involved; NES was generated using software and data bases, such as GSEA, Cytoscape, GO-BP, Reactome and

Hallmark pathways; NES of clusters with similar pathways was averaged to create higher-level groups; cut off for included pathways: P value < 0.05, FDR q value < 0.25. n(sham, radon) = 4. DEG: differentially expressed gene, FC: fold change, GO-BP: gene ontology biological processes, GSEA: Gene set enrichment analysis, NES: normalized enrichment score.

The pathway enrichment analysis showed association of regulated genes with muscle development and activity, collagen and extracellular matrix organization, calcium and calcineurin signaling or transport, potassium and cadmium ion regulation as well as general metabolic and catabolic processes. Also affected are pathways concerning camp signaling, epidermal growth factor and tyrosine kinase activity, endothelial morphogenesis, epithelial maturation, T cell tolerance, keratinization, Ultraviolet (UV) response, and regulation of replication, transcription and translation. Altogether, the regulated signaling pathways indicate that radon exposures affect in skin of CD11c-IL-17A$^{ind/ind}$ mice mainly the organization and maturation of different skin compartments, ion transport and regulation, kinases and T cells, biosynthesis, as well as processes associated with UV response.

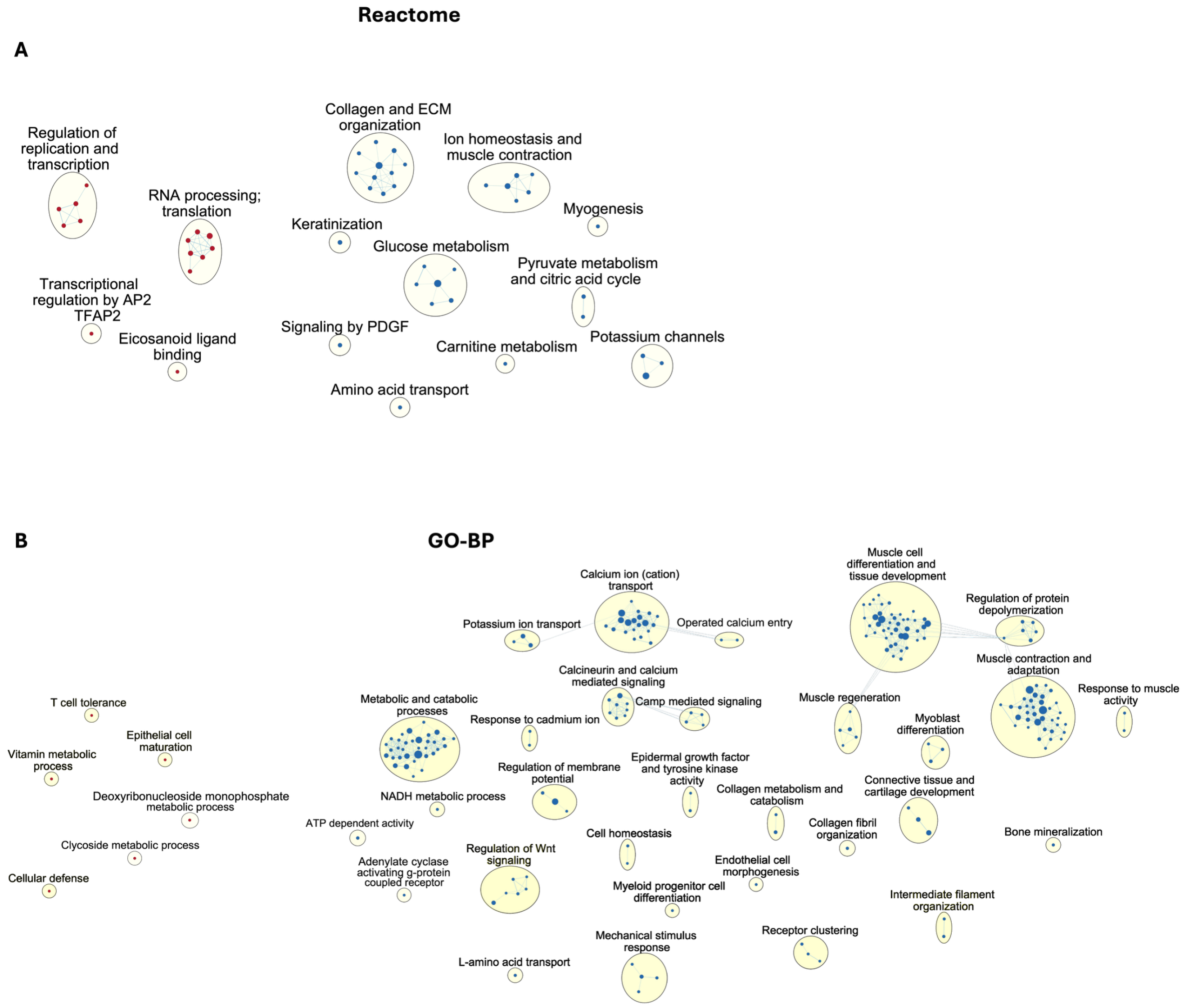

Supplementary Figure S7: Higher-level pathway groups were created after enrichment analysis of transcriptome profiling of non-lesional back skin of CD11c-IL17A$^{ind/ind}$ mice. GSEA of MSigDB Hallmark-, GO-biological process (BP) and Reactome-pathways to identify pathways containing several significant up- or downregulated genes 3 days after 10 consecutive radon exposures with an activity concentration of 39.2 ± 2.0 kBq/m³ (1 hour each) as compared to sham treatment in non-lesional back skin of CD11c-IL-17A$^{ind/ind}$ mice. (A-B) Higher-level enrichment maps created with cytoscape software showing pathways identified via (A) Reactome and (B) GO-BP after multiple radon exposure compared with sham exposure. n(sham, radon) = 4; cut off for included pathways: P value < 0.05, FDR q value < 0.25. GO-BP: gene ontology biological processes, GSEA: Gene set enrichment analysis.

2.5 Comparison of Radon and UV exposure on psoriatic phenotype

In order to find out how relevant our results obtained in a transgenic mouse model are for patients, we compared the transcriptome profiling of non-lesional back skin from CD11c-IL-17A$^{ind/ind}$ mice after

radon exposure with gene expression analyses of human psoriatic skin lesions after UV treatment. For this purpose, we focused on the GEO-available datasets of 3 different studies, GSE162998, GSE53431 and GSE93423, with comparable investigations as performed in our study. Similar to our transcriptome profiling in full back skin of CD11c-IL17A$^{ind/ind}$ mice, the gene expression analysis of GSE162998 was conducted in full psoriatic skin of human biopsies one day after UVB irradiation. In the study of GSE53431, a multiple irradiation setup of psoriasis patients was performed as we did in our analysis with the mice. In contrast to the others, GSE93423 is an *in vitro* study analyzing isolated keratinocytes of psoriasis patients after UVB treatment. Nevertheless, this dataset was chosen as comparison because consistently with our analysis of non-lesional back skin of CD11c-IL17A$^{ind/ind}$ mice, uninvolved skin of psoriasis patients was used.

The Venn diagrams in Supplementary Figure S8A illustrate the numbers of differentially expressed genes (DEGs, P<0.05) overlapping in the direction of regulation (up or down) in our transcriptome data and the GEO datasets. Furthermore, the heat map in Supplementary Figure S8B depicts the respective names and control intensity of the genes identical regulated in our experiments and the analysis of GSE162998, GSE53431 or GSE93423.

Analysis of DEGs, that are regulated in a similar manner after both radon and UV exposure, using the databases uniport.org and genecards.org revealed some overlapping effects of the two treatment options on psoriasis relevant pathways. For example, both treatments regulate genes involved in proliferation and differentiation, as well as calcium transport and intracellular calcium concentration. Furthermore, T cell activation, innate immunity, class I MHC antigen presentation and regulation of TGF-beta signaling are regulated by radon and UV exposure. Also, factors involved in MAP-K cascade and ERK signaling are modulated. The relevance of ERK1/2 in the pathomechanism of psoriasis is evident form the fact that they are also targets of glucocorticoids that are administered as standard treatment (1,2).

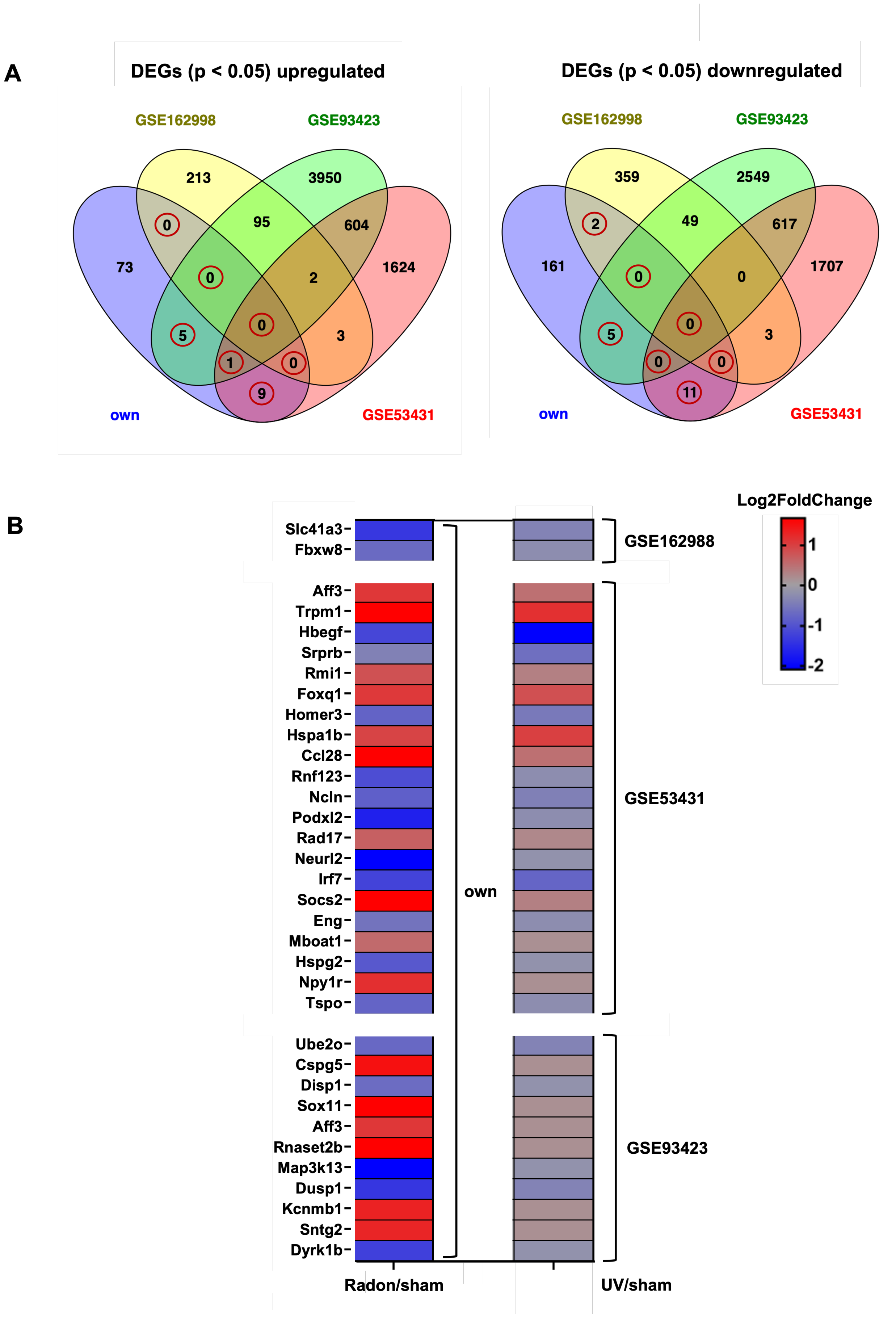
A
DEGs (p < 0.05) upregulated
GSE162998
GSE93423
213
3950
0
95
604
73
0
2
1624
5
0
3
1
0
9
own
GSE53431
DEGs (p < 0.05) downregulated
GSE162998
GSE93423
359
2549
2
49
617
161
0
0
1707
5
0
3
0
0
11
own
GSE53431
B
Log2FoldChange
1
0
-1
-2
Slc41a3
Fbxw8
GSE162988
Aff3
Trpm1
Hbegf
Srprb
Rmi1
Foxq1
Homer3
Hspa1b
Ccl28
Rnf123
Ncln
Podxl2
Rad17
Neurl2
Irf7
Socs2
Eng
Mboat1
Hspg2
Npy1r
Tspo
GSE53431
own
Ube2o
Cspg5
Disp1
Sox11
Aff3
Rnaset2b
Map3k13
Dusp1
Kcnmb1
Sntg2
Dyrk1b
GSE93423
Radon/sham
UV/sham

Supplementary Figure S8: A comparison of DEGs from our transcriptome profiling in non-lesional back skin of CD11c-IL17A$^{ind/ind}$ mice after radon exposures with DEGs form GEO available gene expression analyses of human psoriatic skin lesions after UV treatment shows partially overlapping effects. (A) Venn diagrams show the numbers of DEGs overlapping in direction of regulation in our own dataset and the GEO datasets GSE162988, GSE53431 and GSE93423; left: significantly (P value < 0.05) upregulated genes after radon- or UV-treatment; right: significantly (P value < 0.05) downregulated genes after radon- or UV-treatment; red circles: number of genes overlapping in our own dataset with one or more of the GEO datasets. (B) Heat map represents the gene names and respective control intensity of DEGs regulated in the same direction in our experiments and the GEO datasets GSE162998, GSE53431 and GSE93423; red: significantly upregulated after radon- or UV-treatment as compared to sham group; blue: significantly downregulated after radon- or UV-treatment as compared to sham group; Log2FoldChange indicate the direction of expression regulation. DEG: differentially expressed gene (P<0.05), GEO: gene expression omnibus, UV: ultraviolet.

3 Supplementary References

1. Sevilla LM, Pérez P. Glucocorticoids and Glucocorticoid-Induced-Leucine-Zipper (GILZ) in Psoriasis. Front Immunol. 2019;10(September):1–9.

2. Uva L, Miguel D, Pinheiro C, Antunes J, Cruz D, Ferreira J, et al. Mechanisms of action of topical corticosteroids in psoriasis. Int J Endocrinol. 2012;2012(iv).
